# On Tartrite of Iron and Ammonia

**Published:** 1831

**Authors:** 


### ON TARTRITE OF IRON AND AMMONIA. By MR. AIKIN.

THE following communication we have taken from the Medical Gazette, No. 187.

"Having lately paid some attention to several of the combinations of tartaric acid with oxide of iron and alkali, I beg leave to mention one or two salts but imperfectly known, which appear to me to supply what is yet a desideratum in pharmacy—namely, a soluble salt of iron of uniform composition, not decomposable by alkalies, nor depositing oxyde by keeping, and of no unpalatable taste. In a chemical point of view, too, some of these compound salts are not uninteresting.

It is well known to chemists, being, I believe, first noticed by Klaproth, (Essays, vol. ii. p. 108), that the presence of a certain proportion of tartaric acid in any of the acid solutions of iron entirely prevents the precipitation of the metallic oxyde by the addition of any alkali, pure or carbonated, and in any excess: so that, in fact, there is no method of extracting the oxyde of iron from these compounds, except by calcining the salt in a red heat, to destroy the vegetable acid and then dissolving out the iron from the carbonaceous residue.

The salt which I principally wish to introduce to notice, is the *tartrite of iron and ammonia*, which is made by combining the *proto-tartrite of iron* (itself insoluble) with ammonia, which gives it solubility and all the other properties which may be required for its medicinal use.

When a bundle of fine iron-wire is digested with a warm solution of tartaric acid, hydrogen gas is soon given out; and after a while, the wire becomes coated with a grey crust of proto-tartrite of iron, which partially dissolves in the liquid, so long as there is much excess of acid, giving it a chalybeate taste. But as saturation advances the crust becomes less soluble, and closely adheres to the undissolved iron, so as to make the process of saturation intolerably tedious if left to itself. A little variety of management, therefore, is required, and this may be done in two ways, as follows:—

1. Put into a large iron ladle two ounces of tartaric acid, about as much clean iron filings free from brass, and four ounces of water, and heat it over a fire. Sulphuretted hydrogen gas rises immediately; the mixture soon swells, froths, and thickens, with a slate-grey pasty mass, which begins to form, and the whole must be kept constantly stirred with a broad spatula from beginning to end. More water must be added, which should be no more than to prevent the materials from spirting out by the bubbles of gas, keeping the mixture thick enough to prevent the filings from sinking. In this way, when about twelve ounces of water have been expended, in half an hour the ladle will be filled with a slate-grey puffy mass, scarcely sour to the taste, which may now be emptied into an earthen mortar; and by standing a few hours in a warm place the acidity is entirely gone, and the mixture now consists of grey *proto-tartrite of iron*, mingled with the undissolved filings.

2. Another method, which takes longer time, but saves the wear of the iron ladle, is simply this :—Put into a large earthen mortar any quantity of tartaric acid, with two or three times its weight of iron filings; which, indeed, is much more than is wanted to saturate the acid; but the roughness of the filings is of essential use in mechanically separating the insoluble tartrite when rubbed with the pestle, and thus presenting clean surfaces to the acid. Add hot water, just enough to dissolve the acid, and set it in a warm place to digest. As before, sulphuretted hydrogen rises, together with that black oily fetid scum which always attends the solution of iron when this gas is evolved. In an hour or two some of the grey prototartrite is perceived, and the mass thickens and froths. Continue the digestion in a warm place, rubbing the materials very frequently, and adding just water enough to allow the gas to escape. In this way, with very little pains, the acid becomes saturated in about two days, all sourness disappears, and, as in the former instance, the mixture consists of the grey *prototartrite of iron*, with a large excess of undissolved filings.

Next, without drying the mixture, pour into the mortar some liquid caustic ammonia, the stronger the better, and mix with rubbing. The moment the ammonia touches the prototartrite it changes it to a deep olive green, and the mass, by rubbing, stiffens to the consistence of printers' ink. On further dilution with water (cold distilled), and supersaturation with the alkali, the whole dissolves into a deep bottle-green liquid, leaving little else but the untouched iron filings; which last may be washed with water, dried, and set by for future use.

The green liquid is now a strong solution of ammoniacal prototartrite of iron, with some excess of alkali, and its taste is saline and chalybeate, but without any astringency; and it is by the repeated evaporation and solution of this liquid that the salt is obtained which I propose for use. Put the green liquid, with the washings, into a flat porcelain dish, and evaporate, with a moderate heat, to perfect dryness, but without scorching. As the excess of ammonia flies off the liquid loses its green hue, becomes of a deep red-brown, and yields a salt of the same colour. Redissolve this in water, with a little more ammonia, separate by subsidence and the filter a small portion of brown sediment, and once more evaporate the clear solution to perfect dryness, in a heat about equal to that of a pretty warm oven. Till absolutely dry, the salt is extremely tough and tenacious; but when no more moisture remains it is perfectly brittle, and separates from the vessel with extreme ease.

This salt, the *tartrite of ammonia and iron*, has now the following properties: it is in very shining, glossy, brittle fragments, of so deep a red as to appear black in mass, and looking not unlike crushed garnets, but without any definable crystalline texture. When a little is spread upon paper, in half a minute many of the smaller particles begin to split and fly asunder, like recently fused glass of borax. It is not deliquescent. It dissolves with the greatest ease in water, hot or cold, and almost to any extent, forming a deep red liquid, which yields the salt again on evaporation, unchanged, and without any further deposition of oxide, and this for any number of times. But the most remarkable change that takes place is in the taste. Before evaporation, this was simply saline and chalybeate, but now it has become so strongly saccharine as to equal that of extract of liquorice, and is so powerful when in moderate dilution, as to cover almost every other flavour. Spirit of wine coagulates a strong solution of the salt, but it becomes clear again on dilution. The watery solution pretty soon becomes mouldy when kept by itself, and then it deposits much oxide of iron; but this change may be entirely prevented by adding from a sixth to an eighth of spirit of wine. I have now kept, for a considerable time, a solution of the salt (32 grains to the fluid ounce) in a mixture of seven parts of water and one of spirit of wine, without the smallest change.

This solution, which might be adopted for medicinal use, mixes without visible decomposition with all the alkalies, pure or carbonated, and in any excess; with most of the neutral salts used in medicine—sulphate of soda or of magnesia, for example; with the decoctum aloe c. and with the infusions of orange-peel, quassia, or camomile. The fixed alkalies, however, so far decompose it as to render the ammonia sensible to the taste, but the solution retains its clearness.

With regard to the chemical composition of this salt, it is somewhat complicated, and I have not been able to analyze it fully. It is composed of tartaric acid, ammonia, protoxide and peroxide of iron, and a good deal of saccharine matter. This last is doubtless formed at the expense of part of the tartar, and perhaps by the agency of the protoxide of iron, assisted by a heat above that of boiling water. Hence it is necessary, to form the sugar, that the solution should be fully dried, for the saccharine taste does not appear till then. Acetate of lead causes a copious separation of tartrite of lead, not white, as might be expected, but deeply tinged with oxide of iron, that falls with it. With regard to the state of oxidation of the iron, as it strikes an immediate black with galls, part of the metal must be peroxide, but certainly not the whole, for there is a compound of *pertartrite* of iron and ammonia, which I shall presently describe, which differs essentially from this salt, and which may be converted into it by digestion with fresh iron filings. I know of no means of estimating the relative proportions of these two oxides in a compound like this, but doubtless it is due to the presence of the saccharine matter that the salt makes no farther progress in oxidation during an indefinite number of solutions and evaporations. The mere quantity of metal is easily obtained by calcination. When the dry salt is heated with a spirit lamp on a thin platina shell, it first gives out much vapour of ammonia, with visible dark smoke; it then emits sparks till red hot, when it still retains its form, but with the loss of two-thirds of its weight, and becomes a dark glossy coal, strongly magnetic. By thus igniting 12·5 grains of the salt, I obtained, in two experiments, 4·45 of this magnetic coal, which was deflagrated with a pinch of nitre, dissolved in strong muriatic acid, recovered by ammonia, and gave 4·3 grains of dry red peroxide of iron. Hence we may conclude that 100 grains of the salt contain 20·08 grains of iron, which would make 30·96 of protoxide, or 34·4 of peroxide. Also, 100 parts of this salt contain as much metallic iron as 119 of crystallized sulphate of iron; and these proportions may be borne in mind in prescription for medicinal use.

On the average of several experiments, I find that 100 parts of crystallized acid of tartar yield from 156 to 160 of this tartrite of ammonia and iron.

It is not absolutely necessary that the ammonia be caustic in preparing this salt; the carbonate, I find, will answer very well, but it requires a longer digestion before the grey prototartrite will combine with sufficient ammonia to become entirely soluble. Similar triple salts might doubtless be made by saturating the prototartrite with potash or soda, but there is this advantage in the ammonia, that no nicety of proportion of it is required, as any excess flies off as the solution dries.

I shall now say a few words on the combination of tartaric acid with peroxide of iron and ammonia. When a small quantity of peroxide of iron, wet, and newly precipitated from its muriatic solution by ammonia, is thrown into a hot solution of tartaric acid, it soon dissolves into a red, acid, astringent liquid. More of the peroxide, however, causes most of what is dissolved to separate, and the whole becomes a reddish white subsalt. This, like the prototartrite, is readily made soluble by saturation with ammonia, and is not disturbed by any excess of alkali. When thoroughly dry, it becomes a very tough tenacious mass, totally different from the other in appearance, and extremely absorbent of the moisture of the air, though not entirely deliquescent. To the taste it is saline and chalybeate, but scarcely, if at all, saccharine. Its solution, boiled for some time with iron filings, lets fall much oxide; and the clear liquor, by evaporation, passes into the state of the tartrite before described, with its distinguishing liquorice taste and glossy brittleness.

Many other mixtures still more complicated may be produced by taking a salt of iron already formed, adding tartaric acid, and then saturating the whole with ammonia. All these, if enough of the tartaric acid is present, remain undisturbed by excess of alkali. One compound alone I shall now notice, as I think that this also might be usefully employed in medicine—it is the *tartarized sulphate of iron and ammonia.*

Dissolve green sulphate of iron with half its weight of acid of tartar, in a little cold water; add liquid ammonia, which instantly makes it green and turbid, but the solution becomes quite clear when saturated with the alkali. Evaporate to dryness; redissolve with a little more ammonia, filter if required, and again dry. This forms a deep-red saline mass, readily and totally soluble in water. The taste is saline and chalybeate, but scarcely saccharine. The solution, in mere water, appears to keep for an indefinite time without the smallest change, the tartar being preserved by the well known antiseptic power of the sulphate of iron. This mixes uniformly with alkalies and neutral salts, but is immediately curdled by spirit of wine. When dissolved in water, in the proportion of two or three grains to the ounce, and with a minute excess of soda, the taste is hardly perceptible. In this case also the iron is probably a mixture of the two oxydes. I find that 100 grains of clean sulphate of iron, besides losing its water of crystallization, acquire an increase of about twenty grains by this treatment; so that, in prescription, twelve grains of the dry tartarized sulphate of iron may be considered as containing ten grains of sulphate of iron, taking the latter in its usual crystallized state.

With this salt may be made a convenient *syrupus ferri.* Dissolve eight scruples of the above tartarized sulphate of iron in 2½ ounces of water; melt it in four ounces (Troy) of white sugar, and boil for a few minutes. This yields about five ounces of a brown clear syrup, strongly chalybeate, but not unpalatable when properly diluted; and it does not seem liable to ferment. One fluid drachm of this syrup contains four grains of the tartarized sulphate, equal to 3½ grains of sulphate of iron.

I have also made some *tartarized muriates of iron* in the same way, which have the same general properties, but do not yield results of any particular interest; and the pungent saline taste of the muriate of ammonia predominates so as to render them less palatable than the others.

I remain, Sir,
Yours with respect,
C. R. Aikin."

